# Neuropsychiatric symptoms are related to CSF amyloid and tau in individuals with subjective cognitive decline and mild cognitive impairment

**DOI:** 10.1002/alz.086261

**Published:** 2025-01-03

**Authors:** Ana Luiza Gonçalves Rochetti, Isadora Cristina Ribeiro, Ítalo Karmann Aventurato, Marjorie Cristina Rocha da SIlva, Brenda Costa Gonçalves, Liara Rizzi, Marcio Luiz Figueredo Balthazar

**Affiliations:** ^1^ UNICAMP ‐ UNIVERSIDADE ESTADUAL DE CAMPINAS, CAMPINAS, SP Brazil; ^2^ Universidade Estadual de Campinas (UNICAMP), Campinas, SP Brazil; ^3^ University of Campinas (Unicamp), Campinas Brazil

## Abstract

**Background:**

Alzheimer’s disease (AD) is a neurodegenerative disorder that has become increasingly prevalent around the world and can be characterized in vivo by amyloid‐beta peptide and hyperphosphorylated tau protein. Subjective cognitive decline (SCD) and mild cognitive impairment (MCI) are potential previous stages of AD dementia. Behavioral and psychological symptoms are common in SCD and MCI, but their biological basis is still not clarified. This study aims to investigate the potential correlations between neuropsychiatric symptoms and AD biomarkers in patients with SCD and MCI.

**Methods:**

71 subjects were recruited for a medical and psychological evaluation in the Clinical Research Center of UNICAMP, Brazil. They were diagnosed according to NIA‐AA criteria and underwent neuropsychiatric scales, including the Mild Behavioral Impairment Checklist (MBI‐C), a helpful questionnaire to measure behavioral and neuropsychiatric symptoms in the AD continuum. The subjects also underwent CSF collection to measure the biomarkers in the samples using immunoassay kits. We used Pearson correlation for the statistical analyses.

**Results:**

We found statistically relevant correlations between the MBI‐C C domain and the CSF amyloid‐beta (p = 0.026, r = ‐0.302, n = 54) and a correlation between the MBI‐C E domain and the CSF total‐tau protein (p = 0.026, r = 0.295, n = 57).

**Conclusions:**

The MBI‐C C domain approaches symptoms of impulse dyscontrol, and the MBI‐C E domain comes with symptoms of abnormal perception or thought content. We found that psychological symptoms imply an association of these symptoms with the neurodegenerative process due to the correlation with total‐tau protein, a more specific biomarker.

